# Buyang Huanwu decoction ameliorates myocardial injury and attenuates platelet activation by regulating the PI3 kinase/Rap1/integrin α(IIb)β(3) pathway

**DOI:** 10.1186/s13020-024-00976-0

**Published:** 2024-08-19

**Authors:** Jiaming Gao, Hao Guo, Junmei Li, Min Zhan, Yue You, Gaojie Xin, Zixin Liu, Xiaodi Fan, Qinghe Gao, Jianxun Liu, Yehao Zhang, Jianhua Fu

**Affiliations:** Institute of Basic Medical Sciences of Xiyuan Hospital, China Academy of Chinese Medical Sciences, Beijing Key Laboratory of Pharmacology of Chinese Materia, Courtyard No. 1, Xiyuan Playground, Haidian District, Beijing, China

**Keywords:** Buyang Huanwu decoction, Platelet transcriptomics, Rap1 signaling pathway, Platelet hyperreactivity

## Abstract

**Background:**

Buyang Huanwu Decoction (BYHWD) is a traditional Chinese medicine to treat the syndrome of qi deficiency and blood stasis. Platelets play an important role in regulating thrombus and inflammation after ischemic injury, studies have shown that BYHWD regulate myocardial fibrosis and exert anti-inflammatory effects through IL-17 and TLR4 pathways, but the mechanism of platelet activation by BYHWD in stable coronary heart disease is still unknown. In the present study, model of left anterior descending coronary artery ligation was applied to investigate the mechanisms of BYHWD on modulating platelets hyperreactivity and heart function after fibrosis of ischemic myocardial infarction (MI).

**Methods:**

Myocardial infarction model was constructed by ligation of the left anterior descending coronary artery. The rats were randomly divided into five groups: sham, model, MI with aspirin (positive), MI with a low dosage of BYHWD (BYHWD-ld) and MI with a high dosage of BYHWD (BYHWD-hd) for 28 days.

**Results:**

Coronary artery ligation prominently induced left ventricle dysfunction, increased cardiomyocyte fibrosis, which was accompanied by platelets with hyperreactivity, and high levels of inflammatory factors. BYHWD obviously reversed cardiac dysfunction and fibrosis, increased the thickness of the left ventricular wall, and inhibited aggregation ratio and CD62p expression. BYHWD restored the mitochondrial respiration of platelets after MI, concomitant with an increased telomere expression and decreased inflammation. According to the result of transcriptome sequencing, we found that 106 differentially expressed genes compared model with BYHWD treatment. Enrichment analysis screened out the Ras-related protein Rap-1 (Rap1) signaling pathway and platelet activation biological function. Quantitative real-time PCR and Western blotting were applied to found that BYHWD reduced the expression of Rap1/PI3K-Akt/Src-CDC42 genes and attenuated the overactivity of PI3 kinase/Rap1/integrin α(IIb)β(3) pathway.

**Conclusion:**

BYHWD reduced inflammation and platelet activation via the PI3 kinase/Rap1/integrin α(IIb)β(3) pathway and improved heart function after MI.

**Graphical Abstract:**

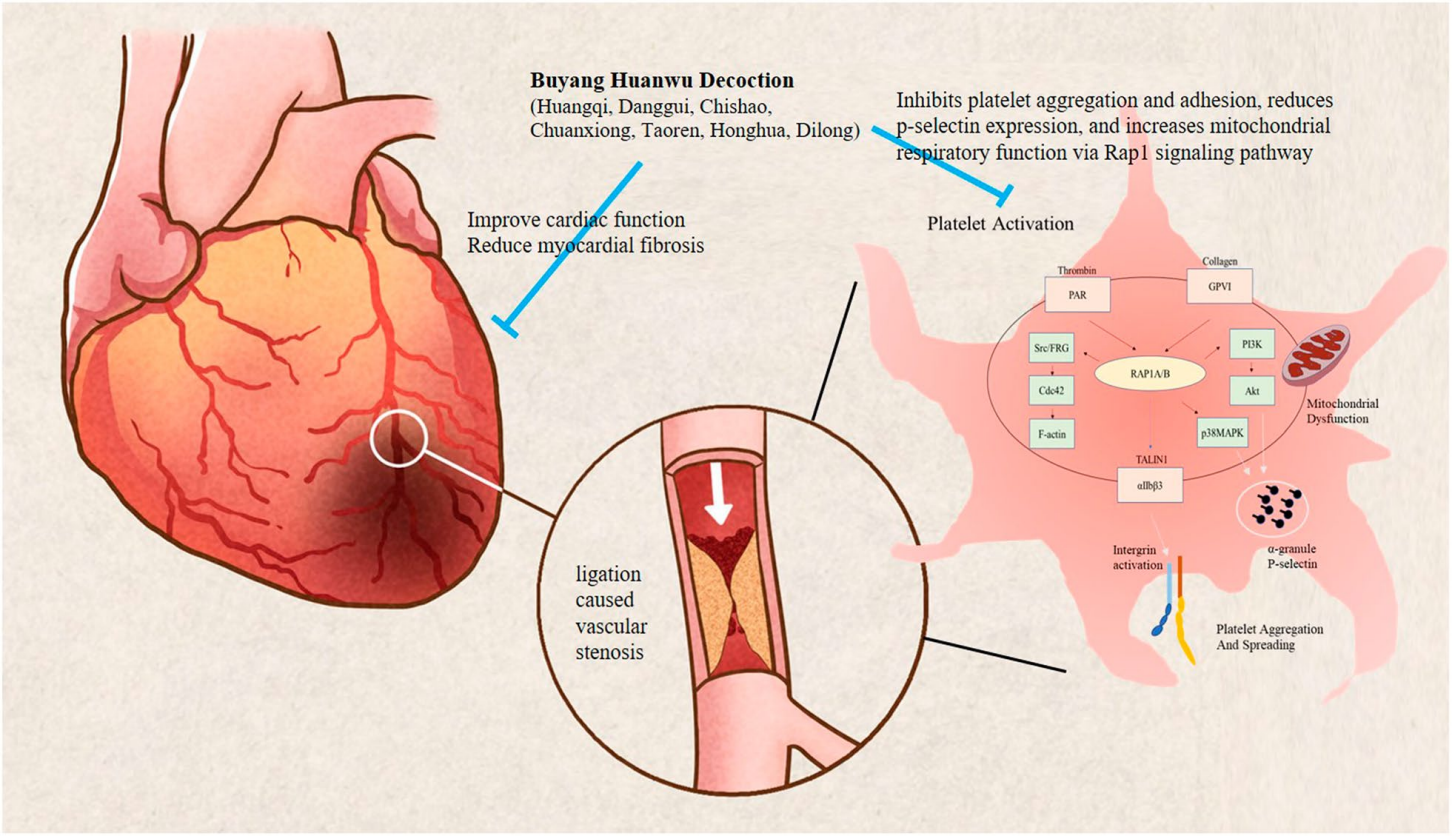

**Supplementary Information:**

The online version contains supplementary material available at 10.1186/s13020-024-00976-0.

## Introduction

Up to date, cardiovascular disease (CVD) has remained one of the leading causes of mortality and morbidity across the world. About 50% CVD patients have been found to be relate to ischemic heart disease [1]. A large decrease of blood flow/oxygen supply to the heart in a short period may cause cardiac metabolic disorders, heart dysfunction, myocardial infarction (MI), and even death. Platelets are hyperresponsive due to exposure to fibrinogen and tissue factor resulting from endothelial injury, and platelet aggregation at the site of vascular injury is the underlying pathophysiology of MI and stroke. Despite effective secondary prevention strategies, patients with cardiovascular disease have recurrent events in 5 to 10% of the population each year [2]. Patients with MI must take antithrombotic drugs for a long time. Based on secondary prevention, aspirin was associated with a lower risk of major adverse cardiovascular events by 19% (including a lower risk of cardiovascular death by 9%) compared to placebo [3], and commonly used drugs such as aspirin can lead to the development of tolerance to drugs with an increased risk of bleeding. Rivaroxaban plus aspirin had better cardiovascular outcomes but more major bleeding events [2].

Platelet activation often occurs after MI despite of antiplatelet therapy. Studies have shown that platelet-derived growth factor-AB promotes post-MI cardiac repair by altering the mechanics related with the infarct scar, leading to robust improvements in cardiac functions [4]. Thrombosis and inflammatory development after MI are components of pathological damage. Emerging research suggests the similarity between the immune regulatory effect of platelets and thrombotic function. Platelets not only play a role in the acute thrombotic event during MI, but also initiate and accelerate the inflammatory effect, contributing to the pathogenesis of MI expansion [5]. Platelets are involved in the coregulatory mechanism of thrombosis and inflammation and play a role in hemostasis and regulation of inflammatory factor levels after acute MI. Another study showed that platelet reactivity exerts a pivotal effect on the pathophysiology of the phenomenon named no reflow and supports the point of view that the thromboinflammatory processes lay the basis of no reflow [6].

Buyang Huanwu decoction, recorded in ‘YilinGaicuo’ of the Qing dynasty in 1830, is a traditional Chinese medicine to treat stroke as well as MI, consisting of *Radix Astragali* (Huangqi) 400 g, *Radix Angelicae Sinensis* (Danggui) 40 g, *Radix Paeoniae Rubra* (Chishao) 40 g, *Rhizoma Ligustici Chuanxiong* (Chuanxiong) 20 g, *Salvia miltiorrhiza* (Danshen) 20 g, *Semen Persicae* (Taoren) 20 g, *Flos Carthami* (Honghua) 20 g, as well as *Lumbricus* (Dilong) 50 g. BYHWD is well known for treating the syndrome of qi deficiency & blood stasis and has been used for the improvement of neurological functional recovery in stroke-induced disability in China for centuries [7]. Mounting clinical and experimental reports demonstrated that BYHWD has remarkable treatment effect against cardiovascular diseases. Zhu et al. [8] found that BYHWD could exert cardioprotective effects on the acute MI model by targeting angiogenesis via caveolin-1/vascular endothelial growth factor (VEGF) signaling pathway. The latest clinical trial of BYHWD in the treatment of ischemic heart failure found that the mechanism was significantly related to complement and coagulation cascades and metabolic pathways [9]. BYHWD inhibits inflammatory reactions and improve plaque stability by regulating lipid metabolism through transforming growth factor-β/smad family member 2 signaling pathway to promote peripheral differentiation of Tregs and maintaining the immune balance of CD4^+^T cells [10]. Augmented BYHWD facilitates axonal regeneration after peripheral nerve transection by reducing the level of inflammatory cytokine tumor necrosis factor-α (TNF-α)/interleukin 1β [11]. The main active ingredients of BYHWD in the impact of platelet function are 6-hydroxykaempferol-di-O-glucoside (from Honghua), paeoniflorin (from Chishao), calycosin-7-O-β-d-glucoside (from Huangqi), galloylpaeoniflorin (from Chishao) and formononetin-7-O-β-d-glucoside (from Huangqi) [12]. In a rat model with occluded left anterior descending coronary artery (LAD), we have demonstrated that BYHWD treatment for 4 consecutive weeks after surgery could improve myocardial fibrosis [13]. Despite that, the roles of platelet/pro-inflammatory mediators, or associated signaling for the post-MI protective effect of BYHWD have never been investigated. Therefore, in our study, platelet function experiments and transcriptomic technology were used to clarify the underlying role of BYHWD in the regulation of platelet reactivity after MI, reducing the risk of thrombus, and helping to decrease inflammation, thus protecting against MI.

## Materials and methods

### Animal model

Sprague–Dawley rats (weighted 200–220 g, male), were purchased from the Animal Center of Xiyuan Hospital (Beijing, certificate no. SYXK 2018-0018), and treated complying with Animal Research Committee’s guidelines for the Animal Center of Xiyuan Hospital. Rats were housed on a light/dark cycle (12:12 h) at 20 ± 2 °C with a humidity of 40 ± 5%, and fed standard rat chow & water. There were 12 rats in each group. Furthermore, the surgical procedures and experimental protocol were approved by Xi Yuan Hospital of China Academy of Chinese Medical Sciences (Protocol No. 2022XLC041). Induction of anesthesia in rats was achieved using intraperitoneal injection of sodium pentobarbital (80 mg/kg), maintained by inhalation of isoflurane (1%) and monitored with a pinch on the toe intraoperatively. The rat’s chest was opened between the second and fourth ribs, followed by LAD ligation using silk (4–0). The animals in the sham group received all surgical treatments except ligation.

### High performance liquid chromatography (HPLC) for extracts of BYHWD

#### Drugs and reagents

BYHWD (Drug approval number: B20050029; Batch number: 190203, 190322, 190403) was provided by Jining Huaneng Pharmaceutical Factory Co., Ltd. Salvianolic acid B (batch number: D1516060, content = 94.1%), paeoniflorin (batch number: A1712063, content ≥ 98%), tanshinone IIA (batch number: L1608056, content ≥ 98%), astragaloside IV (batch number: K1728029, content ≥ 98%) was purchased from Aladdin Biochemical Technology Co., Ltd.(Shanghai, China); the reference substance of amygdalin (batch number: Z28A6L2815, content ≥ 98%) was obtained from Shanghai Yuanye Biotechnology Co., Ltd.; M020170606, content ≥ 98%) and hydroxysafflor yellow A (batch number: A1610261, content ≥ 98%) were purchased from Chengdu Refensi Biotechnology Co., Ltd. Acetonitrile (lot number: LOT155898) and methanol (lot number: LOT095592) were chromatographically pure (Fisher Chemical), formic acid (lot number: LOT095224) and acetic acid (lot number: LOT104221) were chromatographically pure (MREDA, Beijing, China), and the experimental water was D11951 ultrapure water of Thermo Fisher Technology Co., Ltd. (Pittsburgh, PA, USA). Acetonitrile, methanol (HPLC grade), phosphoric acid as well as deionized water (analytical grade) were provided by Fisher Scientific (Shanghai, China).

#### Sample preparation

After being dissolved in methanol, the compounds were filtered with the membrane (0.20 μm) for the preparation of mixed standards, and then BYHWD was finely ground for sample solutions. Passing an 80-target quasisieve, the powder was precisely weighed (0.2 g) and then evenly dissolved in methanol (5.0 mL), and sealed with ultrasonic energy (frequency: 40 kHz, power: 200 w) for sixty minutes, followed by centrifugation at 400*g* for 10 min, take 4.5 mL of the supernatant to obtain a fat-soluble methanol extract. After adding 5.0 mL of triple distilled water and mixing with the residue, extract in a 70 °C water bath for 2 h, and centrifuge at 400*g* for 10 min. Subsequently, 4.5 mL of water-soluble extract and fat-soluble methanol extract were mixed in a 5:7 ratio, and diluted 5 times with the mobile phase; finally, filtered through a 0.22 μm organic filter.

#### Conditions for chromatography

To determine the active components in BYHWD, HPLC was used in combination with a binary low pressure gradient pump, a DAD UV detector, a column oven, as well as an automatic sampler (Agilent 1260, USA). Chromatographic separation was achieved on a CAPCELL PAK C18 column (2.0 × 150 mm, 5 μm, SHISEIDO, Japan) maintained at 40 °C. Mobile phase: phase A—5 mM ammonium acetate, 0.1% acetic acid in water, phase B—acetonitrile. The gradient program was set as follows: 0–25 min, 80–90% A; 25–30 min, 70–80% A; 30–35 min, 60–70% A; 35–40 min, 55–60% A; 40–45 min, 50–55% A; 45–60 min, 40–50% A; 60–65 min, remain 10% A. An aliquot of 20 μL sample solution was injected into the HPLC system for analysis, with a detection wavelength of 275 nm and a flow rate of 0.3 mL/min.

### Drug administration

Complying with the guidelines of Manufacturing Practice and Laboratory Practice, BYHWD (batch number: 190403) was produced from Jining Huaneng Pharmaceutical Factory Co., Ltd. (Shandong, China), with its major components and their contents determined by HPLC fingerprinting. BYHWD was dissolved in saline (2 mL) and administered 48 h after MI using a gavage tube with 0.8 g/kg for BYHWD-ld & 1.6 g/kg for BYHWD-hd, followed by administration every 24 h until day 30 [13, 14]. Aspirin dissolved in 2 mL of saline was administered as a positive control at 100 mg/kg [15]. The rats in the sham group were administered equivalent volumes of saline with the model group in the same way. Rats were anesthetized intraperitoneally 1.5 h after the final administration of BYHWD on day 30, followed by the surgical procedure, and the heart was removed for myocardial morphology and component analysis.

### Echocardiography

Echocardiography for the assessment of cardiac function was carried out using a Vevo 2100 Imaging System (Visual Sonics, Toronto, Canada) equipped with a 30 MHz transducer. Rats were anesthetized with isoflurane in oxygen and put on a heated imaging platform, followed by calculation of these parameters: heart rate (HR), ejection function (EF), left ventricular stroke volume (SV), fractional shortening (FS), left ventricular end-systolic dimension (LVIDs), left ventricular end-diastolic dimension (LVIDd), anterior systolic wall thickness (LVAWs), and diastolic anterior wall thickness (LVPWd).

### Hematoxylin–eosin (H&E) and Masson’s trichrome staining

After being collected from each group, the heart samples were fixed in 4% paraformaldehyde (Solarbio, Beijing, China) for twenty-four hours, embedded in paraffin wax, and sectioned into 4–5 µm thick slices, followed by H&E staining. Subsequently, slices were stained with Masson’s trichrome, showing red in normal tissues but blue in collagen tissues. A light microscope (Olympus BX51 microscope, Japan) was applied for high resolution image of heart sections. Quantitative analysis of collagen deposition was achieved using collagen volume fraction (CVF) with the application of Image Pro Plus (Media Cybernetics Inc, MD, USA).

### Electron microscopy of myocardial tissue

Rat hearts were fixed with 2% glutaraldehyde (Solarbio, Beijing, China). Myocardial tissues were collected from the surrounding infarcted region of the left ventricle, cut into blocks (1 mm^3^), fixed overnight at 4 °C, washed for three times with phosphate-buffered solution (0.1 mol/L), and then post-fixed with osmium tetroxide (1%) for 2 h. Subsequently, we routinely prepared the ultrathin sections and photographed them by a transmission electron microscope (JEM 1400 plus, JEOL, Tokyo, Japan).

### Detection of the Luminex liquid suspension chip

It was conducted by using the Bio-Plex Pro Chemokine Panel kit (40-plex) that complies with the instructions of the manufacturer. Specifically, sera with/without BYHWD treatment were incubated in 96-well plates which were embedded with microbeads for 1 h, detection antibody for thirty minutes, and streptavidin–phycoerythrin (PE) for 10 min, followed by detection using the BioPlex MAGPIX System (Bio-Rad).

### Viscosity determination

Blood samples (200 µL) were collected in heparin and used to determine viscosity at different shear rates using a cone-plate viscometer (Model LG-R-80B, Steellex Co., China) at 37 °C. Whole blood viscosity was measured at a low shear rate of 5/S, a medium shear rate of 60/S and a high shear rate of 150/S.

### Platelet number count and platelet isolation

After SD rats were anesthetized with intraperitoneal injection of 1% sodium pentobarbital, blood (10 mL) collection from the abdominal aorta was performed using a tube with acid-citrate-dextrose anti-coagulant (3.2% trisodium citrate, 0.109 mol/L). The ratio of anticoagulant to blood is 1:9. The whole blood was then centrifuged (120*g*, 15 min) at room temperature to obtain platelet-rich plasma (PRP). Subsequently, the PRP supernatant was pelleted and centrifuged (300*g*, 15 min). Finally, platelets were resuspended in modified Tyrode buffer (Leagene Ciotech. Co., Ltd., Beijing, China). Whole blood (20 µL) was diluted with blood diluent buffer (MEK640, NIHON KOHDEN, Japan) and tested on automated hematology analyzer (NIHON KOHDEN CORP., Japan) to calculate the number of platelets.

### Clot retraction

The washed platelets (100 μL) were mixed with platelet poor plasma (PPP, 300 μL) and induced to coagulate with thrombin (0.2 U/mL) [16]. After the incubation at 37 °C for different time intervals, the mixture was photographed and the degree of clot retraction was further analyzed by Image J software (version 1.4.3.67). Retraction (%) = 100 − [(sample clot area/intact sample area) × 100].

### Flow cytometry analysis

Whole blood was incubated with anti-rat CD61 antibody labeled with phycoerythrin (104315) and anti-rat CD62P antibody labeled with PE (148305, BioLegend, California, USA), with the concentration finally reaching up to 20 mg/mL. After incubation for thirty minutes in darkness, the mixture was analyzed using a flow cytometer (LSRFortessa with FASCDiva software; Becton Dickinson, USA). Platelets were identified based on their characteristic light scatter as well as CD61 antibody binding. The activated platelets were calculated using the percentage of 10,000 CD61 platelets to exhibit Allophycocyanin-CD62P fluorescence.

### Platelet aggregation

Fresh platelets were isolated from each group as described above and treated with adenosine diphosphate (ADP) for 15 min. Subsequently, platelet aggregation was detected using a single-channel aggregometer (Chrono-Log Corp, USA) and reported using the percent of aggregation at 3 min and normalized to a standard deflection, corresponding to light transmission through PPP.

### Platelet spreading

As previously described [17], 96-well cell plates were precoated with fibrinogen at 4 °C overnight and then blocked with bovine serum albumin (BSA) (1%, room temperature) for an hour. Subsequently, fresh platelets were diluted using Ca^2+^-free modified Tyrode solution consisting of Mg^2+^ (2 mmol/L) and were spread on immobilized fibrinogen at 37 °C. 200 μL saline control, BYHWD-ld, BYHWD-hd and aspirin were correspondingly added to the wells of each group and incubated for 30 min. Following fixation with PFA (4%), adhesion was stopped and platelets were permeabilized with Labeling solution (0.2% Triton X-100, 0.5% BSA). The adherent platelets were stained with Calcein. Finally, the fluorescence intensity was captured at Ex/Em = 496/515 nm.

### Oxygen consumption rate (OCR)

Fresh platelets from the sham, model, BYHWD-hd and positive groups were extracted by the method described above and used in this assay. PRP was diluted into 10^^^8 per well using PPP. Medium (80 µL) was added to wells B-G, and the same amount of culture medium was added to wells A and H as controls, followed by centrifugation (5 min, at 200*g* and 20 ℃) for cell adhesion. After 30 min, the culture medium was taken place by the same amount of assay medium. Substances, including Oligomycin (10 µM), carbonyl cyanide *p-*trifluoromethoxyphenylhydrazone (FCCP) (0.5 µM), antimycin A along with rotenone (0.5 µM), were added into the kit pack to block the mitochondrial respiration chain complex. Quantification of OCR was performed using an extracellular flux analyzer (Seahorse, Agilent, USA).

### Platelet RNA isolation and RNA sequencing

The platelets from the sham, model, and BYHWD-hd groups were extracted by the method described above and used in this assay. After the samples were treated with Trizol (Invitrogen, California, USA), total RNA was purified, followed by deoxyribonuclease. Quality assessment of the resulting RNA was conducted using a bioanalyzer assay. Based on the Ovation RNA Seq v.2 kits (NuGen, Inc., San Carlos, CA), total RNA (DNA-free) was adopted as input for library construction. Following the final step for amplification, libraries (250- 450 base pairs) were selected. After mixed, barcoded RNA-seq libraries were subjected to 75 base pair paired-end sequencing using an Illumina HiSeq-2000 machine (Illumina, San Diego, California, USA). Additionally, each sample has the equivalent of 1 dedicated flow cell. After demultiplexed, the sequencing data was converted to the format of FASTQ. Paired-end reads were aligned with RefSeq using TopHat2. RSeQC v2.3.9 (Boston, USA) was adopted for the calculation of RNA reads per kilobase per million mapped reads (RPKM). To ensure that the actual transcript levels obtained from the measurements, all the downstream analysis of the RNA-seq data was on the basis of a minimum RPKM threshold (0.3).

### Quantitative real-time PCR (RT-qPCR) for total RNA

Platelets of sham, model, BYHWD-ld, and BYHWD-hd groups were extracted by the method described above and used in this experiment. On the basis of a PrimeScript RT Reagent Kit (Takara, Japan), reverse transcription of total RNA was conducted. RT-qPCR was conducted with the application of QuantStudio™ Test Development Software (Thermo Scientific, USA) as well as SYBR Green RT-qPCR Master Mix (EZBioscience, USA). Besides, data analysis was carried out using the 2 − ΔΔCT method. All primers were purchased from Sangon Biotech (Shanghai, China). Sequences of the primers are listed in Supplementary Material Table 1.

### Telomere quantitative FISH (Q-FISH), immunofluorescence microscopy, and image acquisition

Telomerase activation alleviated interstitial fibrosis in the non-infarcted region of the LV, which significantly reduced the mortality from heart failure after MI [18]. Therefore, we conducted the corresponding tests to explore whether BYHWD alleviates the degree of telomere damage during myocardial infarction. After blocked with calf serum (4%)/Triton X-100 (0.1%)/phosphate buffer saline (PBS), tissues were stained with prediluted troponin-T (cTnT) antibody (ab47003, Abcam) for 2 h at room temperature overnight at 4 °C, then washed with PBS, followed by incubation with 1:400 goat anti-rabbit Alexa 488 (Abcam, Cambridge, UK) for 1 h, and counterstaining with 4',6-diamidino-2-phenylindole (DAPI, 1 μg/mL) in PBS for 5 min, washed with ddH_2_O, air-dried, and then mounted with ProLong Gold Antifade (Life Technologies, Pittsburgh, USA). In addition, the images were captured by an Olympus spinning-disk confocal microscope based on a PLAN APO 40× objective. The measurement of telomere Q-FISH was performed using TelC-Cy3 peptide nucleic acids (PNA) probe (F1002, PNA Bio, Daejeon, South Korea) [19]. A PNA signal that normalized to nuclear DAPI among telomere fluorescence units (TFUs) among cTnT + cardiomyocytes (CMs) were recognized as telomere signal intensity, and then captured using the ImageJ plugin Telometer. Subsequently, telomere levels were blindly scored within 5–6 interested regions for 2 non-consecutive sections. This study uses the method of Chang et al. [19] and the description of the methods partly reproduces their wording.

### Protein extraction and western blot analysis

The whole protein of 50 mg platelet precipitation descried above was extracted using a protein extraction kit (Applygen Technologies, Beijing, China) complying with the instructions of the manufacturer, separated on SDS polyacrylamide gel electrophoresis (10%) and transferred to the membrane of polyvinylidene difluoride. Membranes were firstly blocked with 5% BSA dissolved in Tris buffered saline (TBS) with Tween for a hour, and then probed with appropriate antibodies at 1:1000 against phosphoinositide-3-kinase (PI3K) (ab154598, Abcam, Cambridge, UK), phospho-phosphoinositide-3-kinase (p-PI3K) (341468, ZEN BIO, Chengdu, China), AKT serine/threonine kinase (Akt) (ab179463, Abcam, Cambridge, UK), phospho-Akt serine/threonine kinase (p-Akt) (ab38449, Abcam, Cambridge, UK), Rap1(#2399, CST, Boston, USA), cell division cycle 42 (CDC42) (ab187643, Abcam, Cambridge, UK), nonreceptor tyrosine kinase (Src) (2019S, CST, Boston, USA), phospho-nonreceptor tyrosine kinase (p-Src) (6943S, CST, Boston, USA), beta-actin (ab6276, Abcam, Cambridge, UK) at 4 ℃ with gentle agitation overnight. After incubation with horseradish peroxidase-conjugated IgG (1:10,000) as secondary antibody for 1 h at room temperature, blots were detected using an enhanced chemiluminescence detection system.

### Statistical analysis

Statistical analysis was conducted using GraphPad Prism 7 software (GraphPad software Inc., USA). All data were described with means ± standard deviation (SD). Comparisons for groups was performed using one-way ANOVA, followed by Bonferroni test. A P value less than 0.05 indicates statistical significance.

## Results

### Identification of the main chemical ingredients of BYHWD

**Fig. 1:**
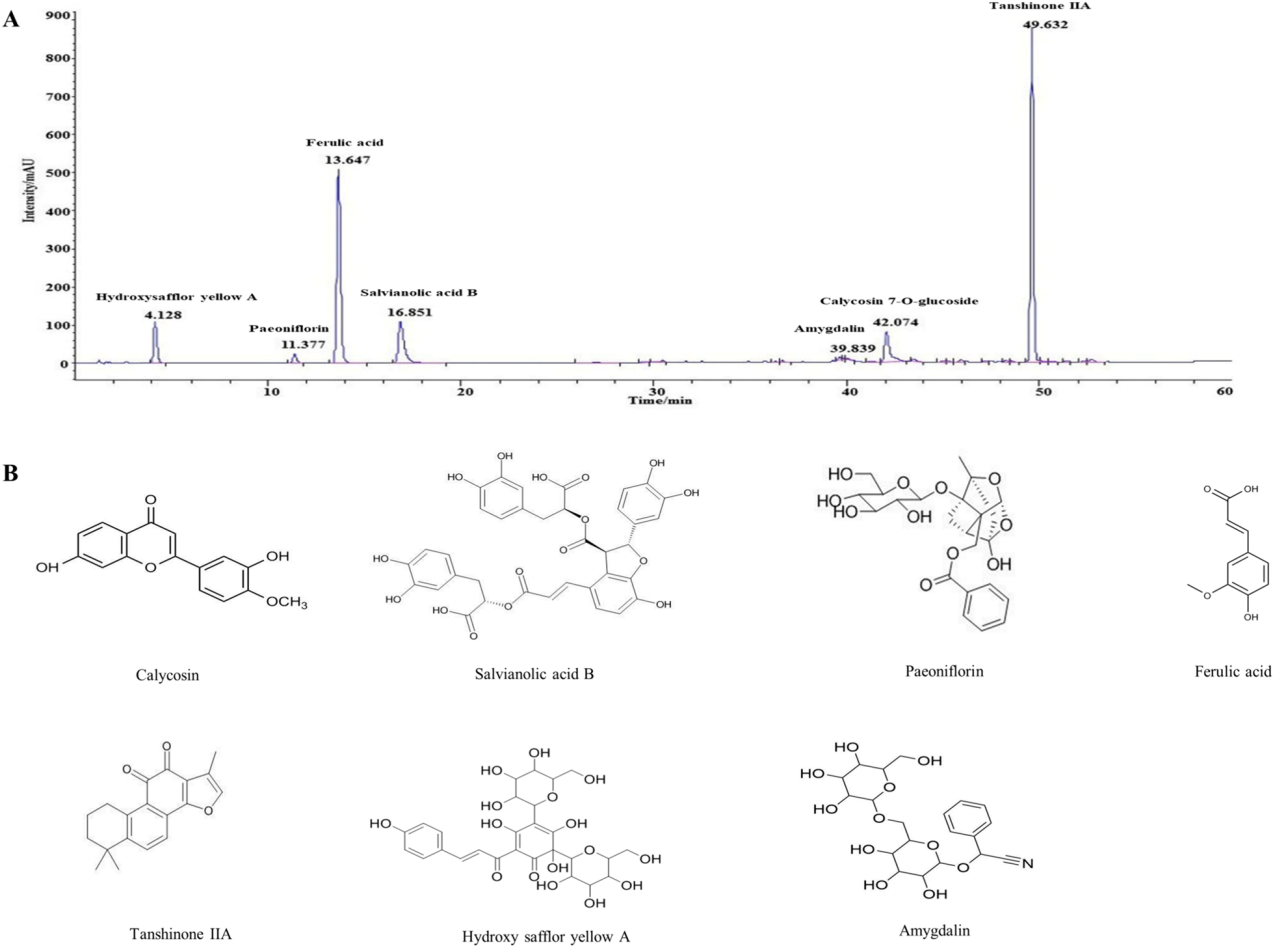
Identification of the main chemical components in BYHWD. **A** The water-soluble and methanol-soluble components of BYHWD were determined by HPLC at 275 nm. The mobile phase consists of ammonium acetate (5 mM), aqueous solution of acetic acid (0.1%) as well as acetonitrile. **B** Chemical structures of 7 main components of BYHWD

Compared to the standards, a total of 7 chemical ingredients of BYHWD were identified and quantified, which were Hydroxysafflor yellow A, Paeoniflorin, Ferulic acid, Salvianolic acid B, Amygdalin, Calycosin 7-O-glucoside and Tanshinone IIA (Fig. 1).

### BYHWD improves cardiac function after ischemic injury and myocardial tissue pathological structure, and reduces myocardial fibrosis

**Fig. 2:**
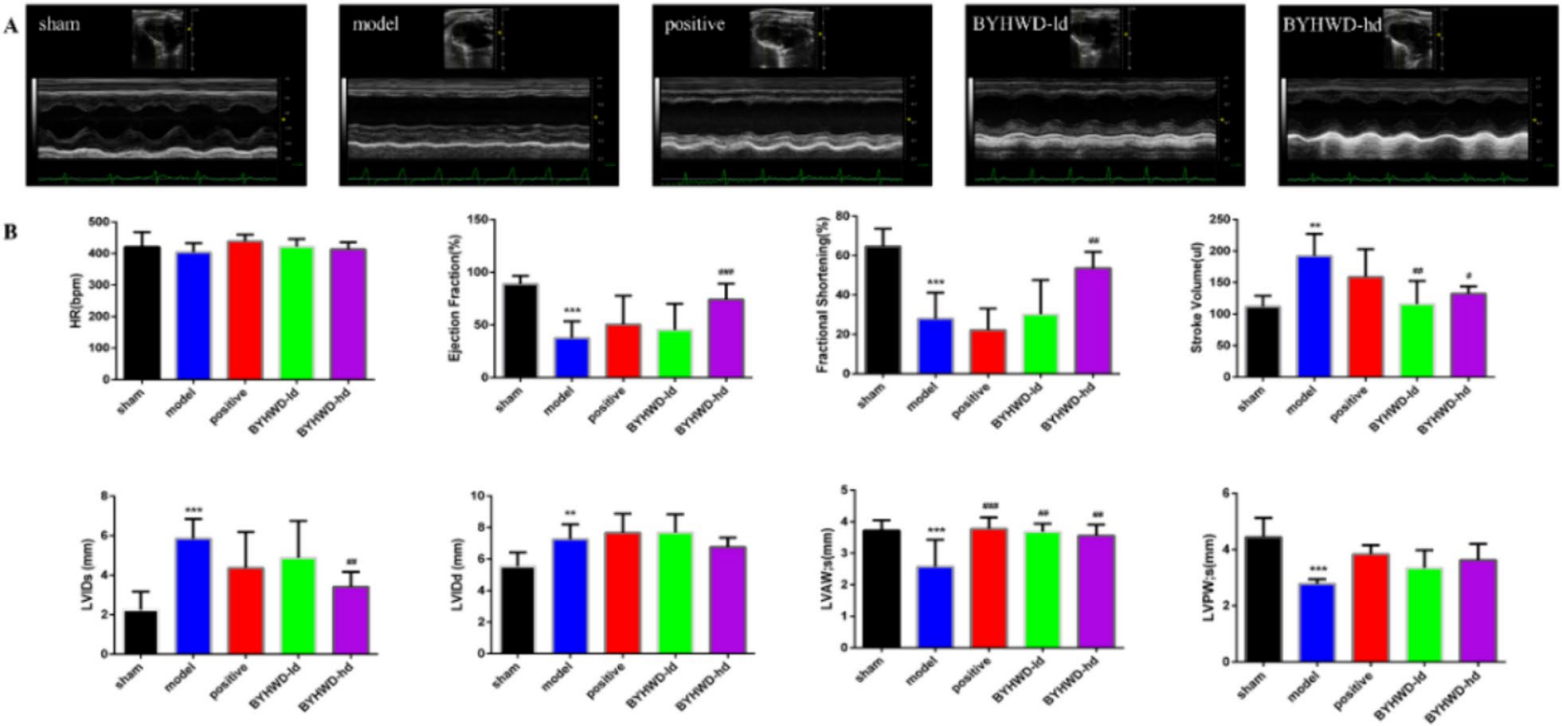
BYHWD improved cardiac function in MI rats. **a** Representative M-mode echocardiography tracings. **b** Quantification parameters for the assessment of cardiac function including heart rate (HR), ejection fraction (EF), stroke volume (SV), fractional shortening (FS), left ventricular end-systolic dimension (LVIDs), left ventricular end diastolic dimension (LVIDd), anterior systolic wall thickness (LVAWs), and diastolic anterior wall thickness (LVPWd). Data are described with mean ± SD (n = 6 in each group). ^*^P < 0.05 and ^**^P < 0.01, compared to the sham group; ^#^*P* < 0.05 and ^##^*P* < 0.01, compared to the model group

Echocardiography was performed 4 weeks after MI to evaluate cardiac function. The results showed that in contrast to the sham group, significant decreases in functions (such as the values of EF, FS, and SV) of the left ventricle (LV) in the model group were found (Fig. 2). Administration of BYHWD significantly improved cardiac function. Besides, echocardiography (M‐mode) at the border of the infarct zone showed improvements in LV wall movement with statistical significance among rats administered BYHWD in contrast to the model group (Fig. 2), showing equivalent heart rates for the two groups. In addition, the thickness of LV end‐diastolic wall in diastole (LVEDd) was decreased after MI in contrast to the sham group and was attenuated in the rats administered BYHWD to some extent. In particular, an increase in the thickness of anterior systolic wall was found 4 weeks after MI, indicating that a protective overcompensatory mechanism was formed by increasing the thickness of remote LV wall following MI (Fig. 2). Based on the above results, it could be inferred that BYHWD exerts an impact on the improvement of cardiac function following myocardial ischemic injury.

**Fig. 3:**
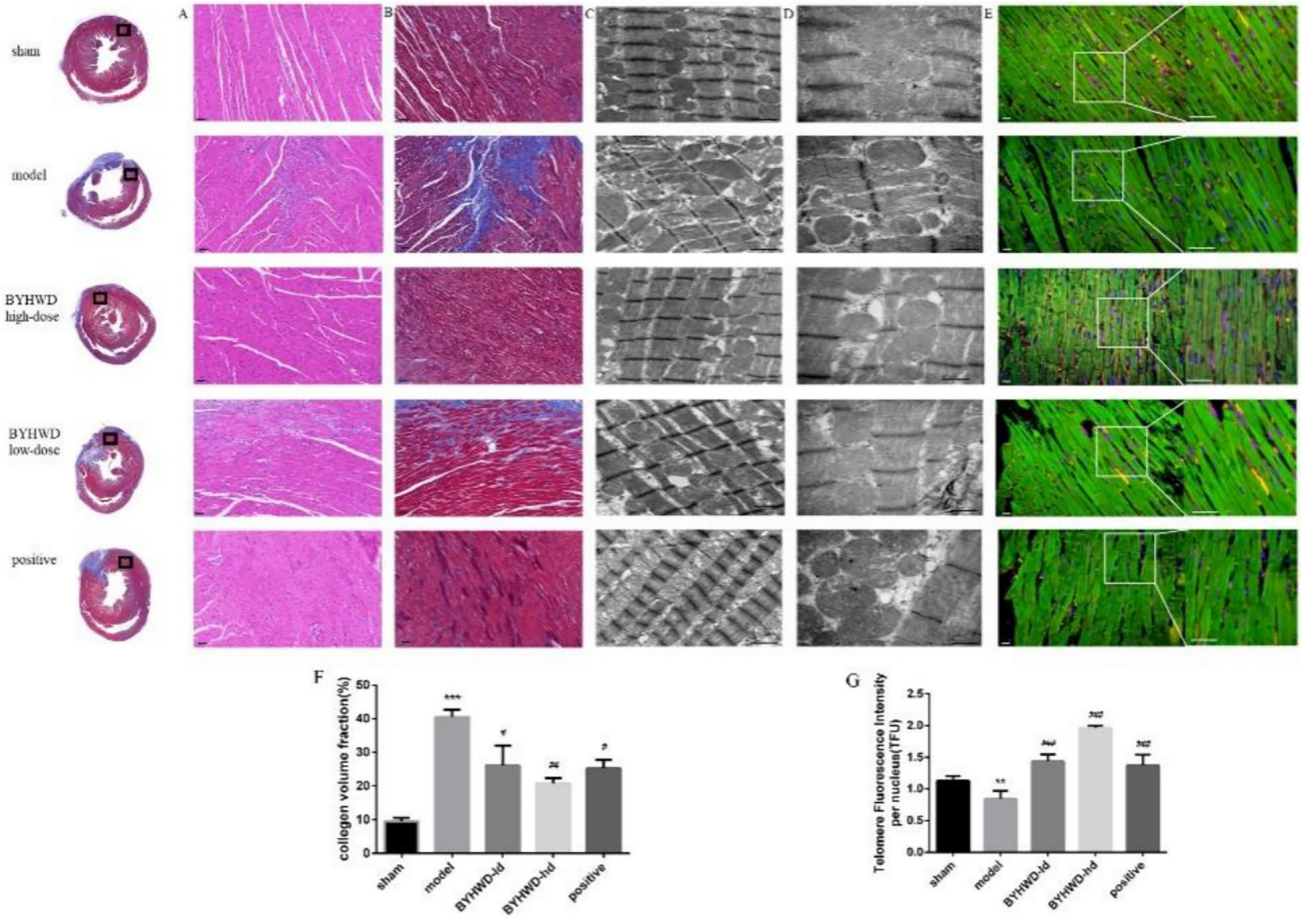
BYHWD attenuated left ventricular myocardial morphological remodeling and fibrosis in MI rats. **a** H&E staining images, scale bar: 50 μm; **b** Representative Masson staining images, scale bar: 50 μm; **c**, **d** Representative images of myocardial tissue under a TE microscope, ×20,000, scale bar: 1 μm, ×40,000, scale bar: 0.5 μm. n = 6 in each group. **e** Cardiomyocytes were stained for cardiac Tn-T (green), telomere (red) as well as nuclear DAPI (blue) in sections of cardiac tissues. Scale bars, 50 μm. **f** Quantification of interstitial cardiac fibrosis. **g** Telomere signal intensity per DAPI-stained nucleus is expressed as mean ± SEM, ^*^P < 0.01, ^**^P < 0.01 and ^***^P < 0.001, compared to the sham group. ^#^*P* < 0.05, ^##^*P* < 0.01, and ^###^*P* < 0.001, compared to the model group

The PFA-fixed heart sections were stained with H&E as well as Masson’s trichrome to observe the pathological conditions of the myocardial tissues. The model group exhibited severe inflammatory infiltration, disordered CM arrangement, and mitochondrial swelling, and the degree of tissue fibrosis around the infarct area was aggravated (Fig. 3A, B). Aspirin could relieve MI to some extent, but the myocardial mitochondria were swollen, the inner membrane structure was dissolved, and the quality of the mitochondria had decreased. BYHWD improved myocardial inflammatory infiltration and myocardial fibrosis around the infarcted area, and the BYHWD-hd group had better improvement effect than the BYHWD-ld group. It also improved the arrangement of myocardial cells, with obvious sarcomeres and intact mitochondrial membrane structures under an electron microscope (Fig. 3C, D). By using Q-FISH, we measured TFUs in CMs. Quantification entailed the measurements using a PNA probe against the telomere repeat normalized to nuclear DAPI in individual CMs. The telomere levels of CMs were significantly reduced by an average of 20% in the model group compared to the sham group. Unlike the model, the BYHWD-ld and BYHWD-hd groups exhibited 25% and 46% increases in telomere levels, respectively (Fig. 3E). The quantitative data on cardiac interstitial fibrosis and TFU were shown in Fig. 3F and G.

### Effects of BYHWD on serum inflammatory factor levels in rats with MI

**Fig. 4:**
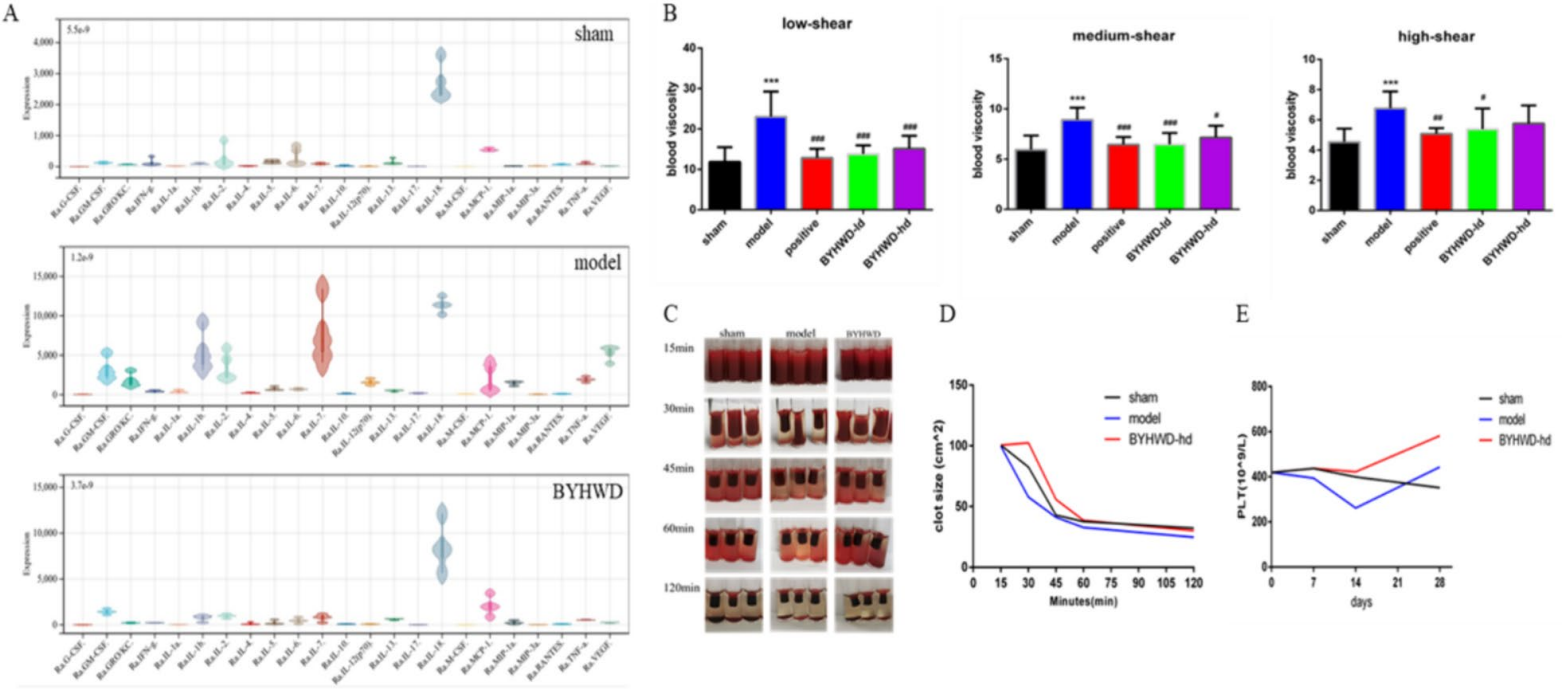
BYHWD regulates plasma inflammatory factors, blood viscosity, and thrombus elasticity in MI rats. **a** Plasma inflammatory factor levels, including IL-6, IL-10, TNF-α, IFN-γ, and VEGF. **b** Plasma viscosity under different shear conditions. **c** Clot retraction within 120 min. **d** Clot retraction quantification value. **e** Platelet number at different time points of MI. ^*^P < 0.05 and ^**^P < 0.01, compared with the sham group; ^#^*P* < 0.05 and ^##^*P* < 0.01, compared to the model group; Data are described with means ± SD; n = 6 in each group

The detection of multiple plasma inflammatory factors revealed that plasma interleukin 6 (IL-6), interleukin 10 (IL-10), TNF-α, interferon gamma (IFN-γ) content was significantly increased in the model group but the levels of inflammatory factors were decreased after the administration of BYHWD. Except for interleukin 18, the other factors concentration were not different from those recorded in the sham group (Fig. 4A).

### The effect of BYHWD on platelet-mediated clot retraction and hemodynamics

Compared to the sham group, the blood viscosity of the model group increased significantly under low, medium, and high shear stress, and both BYHWD and aspirin reduced the blood viscosity (Fig. 4B). The in vitro thrombus retraction experiment showed that the blood clot retracted rapidly in the model group within 15–30 min, and BYHWD significantly reduced the retracted volume of the thrombus within 30–45 min (Fig. 4C, D). With the extension of modeling time, the number of platelets fluctuated, but there was no statistical significance (Fig. 4E).

### BYHWD blocks ADP-induced platelet aggregation and affects P-selectin expression and fibrinogen binding

**Fig. 5:**
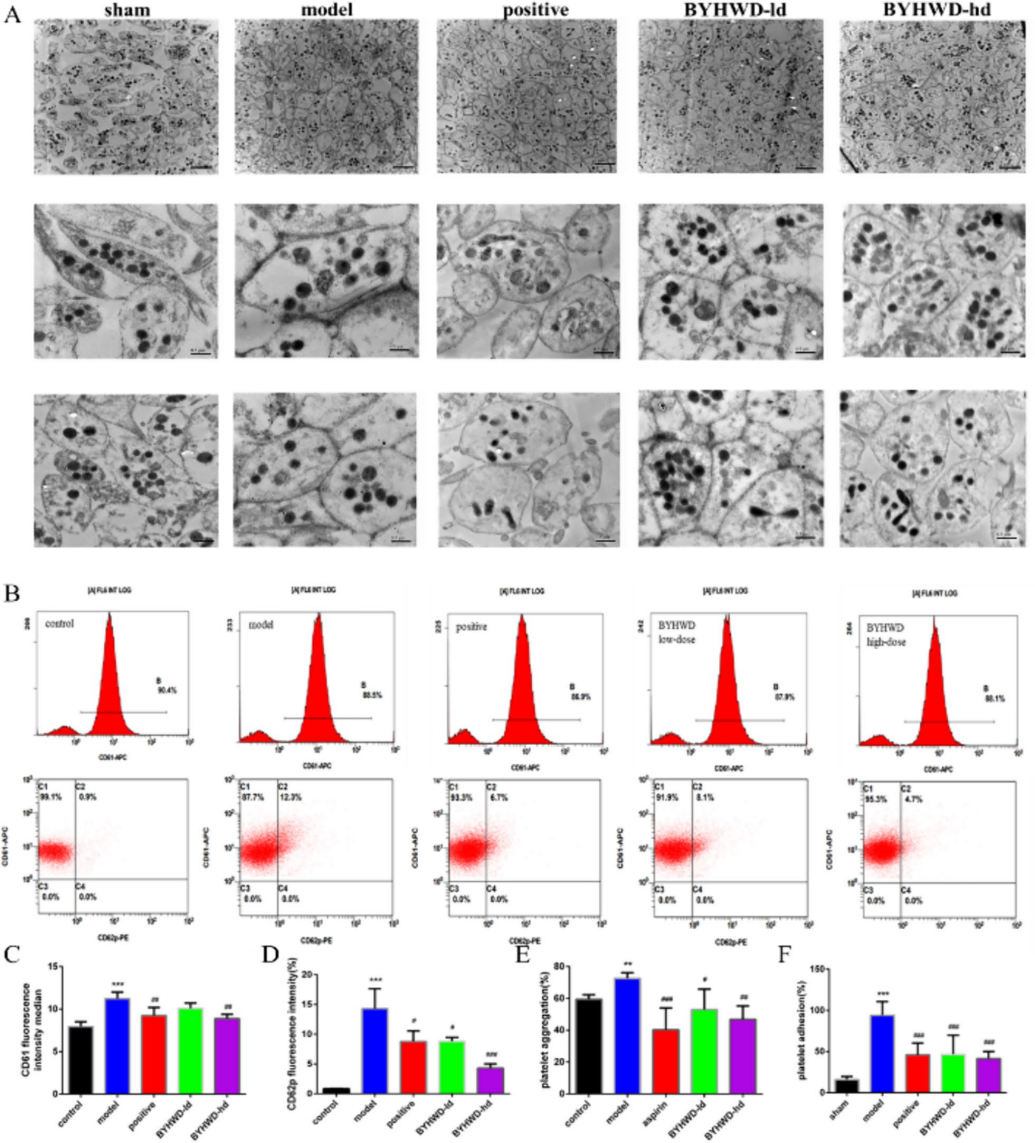
Effect of BYHWD on platelet activation in MI rats. **a** Platelets under a TEM (×8000, scale bar: 2 μm; ×30,000, 0.5 μm). **b** Platelets were labeled with CD61 and CD62p in Flow cytometry. **c** Median statistical results of the CD61 fluorescence values. **d** Statistical results of the CD62p fluorescence intensity. **e** ADP-induced platelet aggregation. **f** Adhesion of platelets to fibrinogen. Data are presented with mean ± SD (n = 6 in each group). ^*^P < 0.05 and ^**^P < 0.01 when compared to the sham group; ^#^*P* < 0.05 and ^##^*P* < 0.01 when compared to the model group

The results of transmission electron microscopy in each group showed a different number of mitochondria (gray) and degranulation (dark) in the BYHWD group compared to the model group with statistical significance. The average cross-section of platelets in the model group was increased and after BYHWD-hd treatment, the average cross-section of platelets was significantly reduced (Fig. 5A). The flow cytometry results showed that BYHWD reduced the expression of the platelet-activating glycoprotein CD62p (Fig. 5B–D) and regulated the deformation caused by platelet activation. The in vitro platelet aggregation experiments revealed that BYHWD reduced the rate of platelet aggregation induced by the ADP agonist (Fig. 5E). In the in vitro adhesion experiment, the platelet–fibrin adhesion rate was increased in the model group. After administration of BYHWD and aspirin, the platelet adhesion rate was significantly reduced (Fig. 5F).

### BYHWD improved platelet mitochondrial function and mitochondrial respiration

**Fig. 6:**
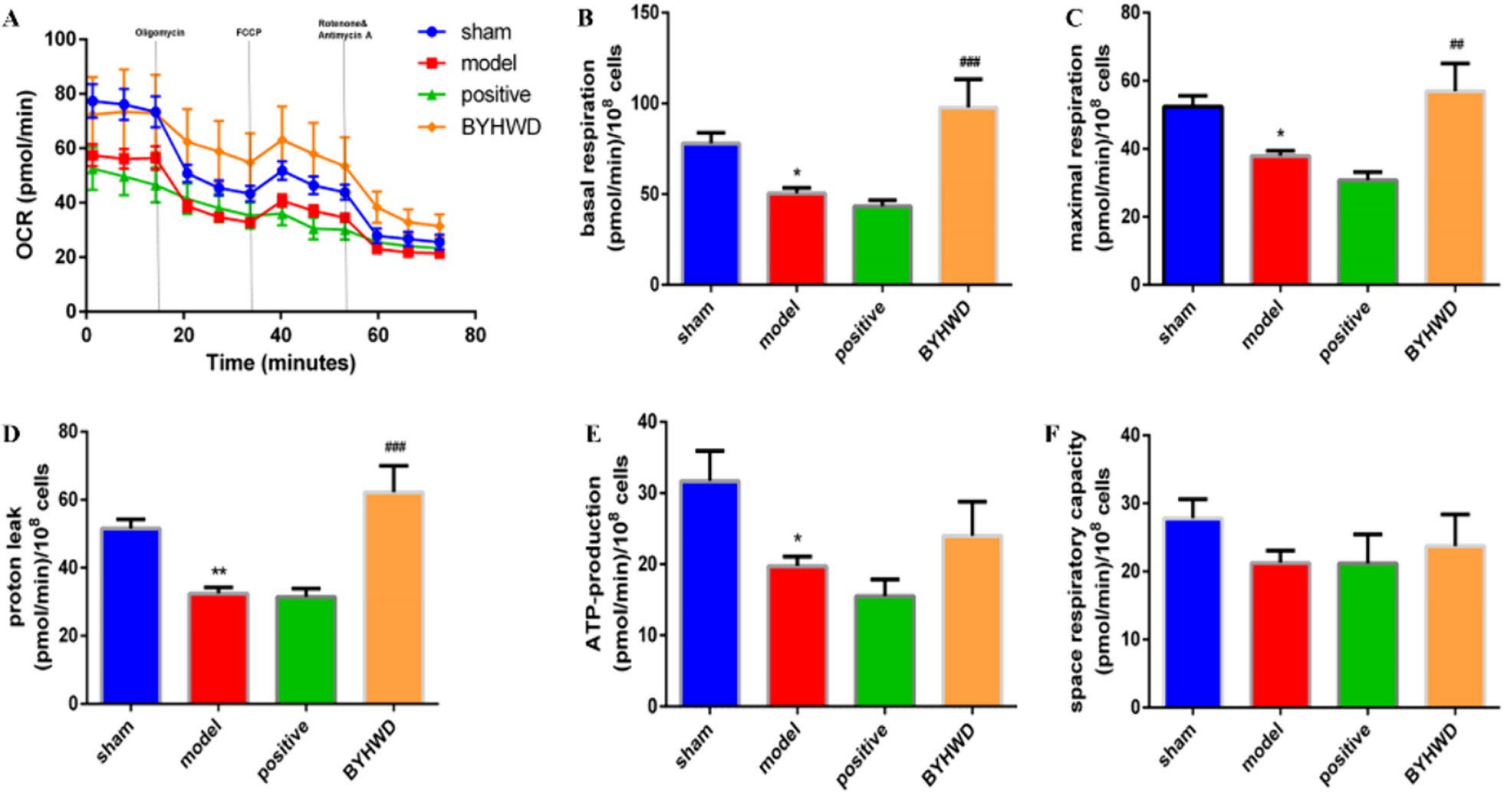
Effects of BYHWD on platelet mitochondrial respiration after MI. **a** Mitochondrial respiration assay of the OCR was performed with a Seahorse XF 96 analyzer. Oligomycin (10 µM), carbonyl cyanide *p-*trifluoromethoxyphenylhydrazone (0.5 µM), antimycin A as well as rotenone (0.5 µM) were sequentially added in order to block the electronic respiratory chain. **b**–**f** Quantitative analyses of mitochondrial respiration parameters, including basal respiration, maximal respiration, proton leakage, adenosine triphosphate (ATP) production, and spare respiratory capacity. Data are presented with mean ± SD (n = 6 in each group). ^*^P < 0.05 and ^**^P < 0.01 when compared to the sham group; ^#^*P* < 0.05 and ^##^*P* < 0.01 when compared to the model group

The mitochondrial stress test experiment showed that mitochondria function in the model group significantly decreased, and the response curve of mitochondria has a noticeable drop. BYHWD-hd increased mitochondrial respiration ability, improved basal and maximal respiration, and increased ion leakage penetration capacity to some extent (Fig. 6).

### BYHWD regulates the expression of proteins and genes related to platelet activation after MI via Rap1 signaling pathway

**Fig. 7:**
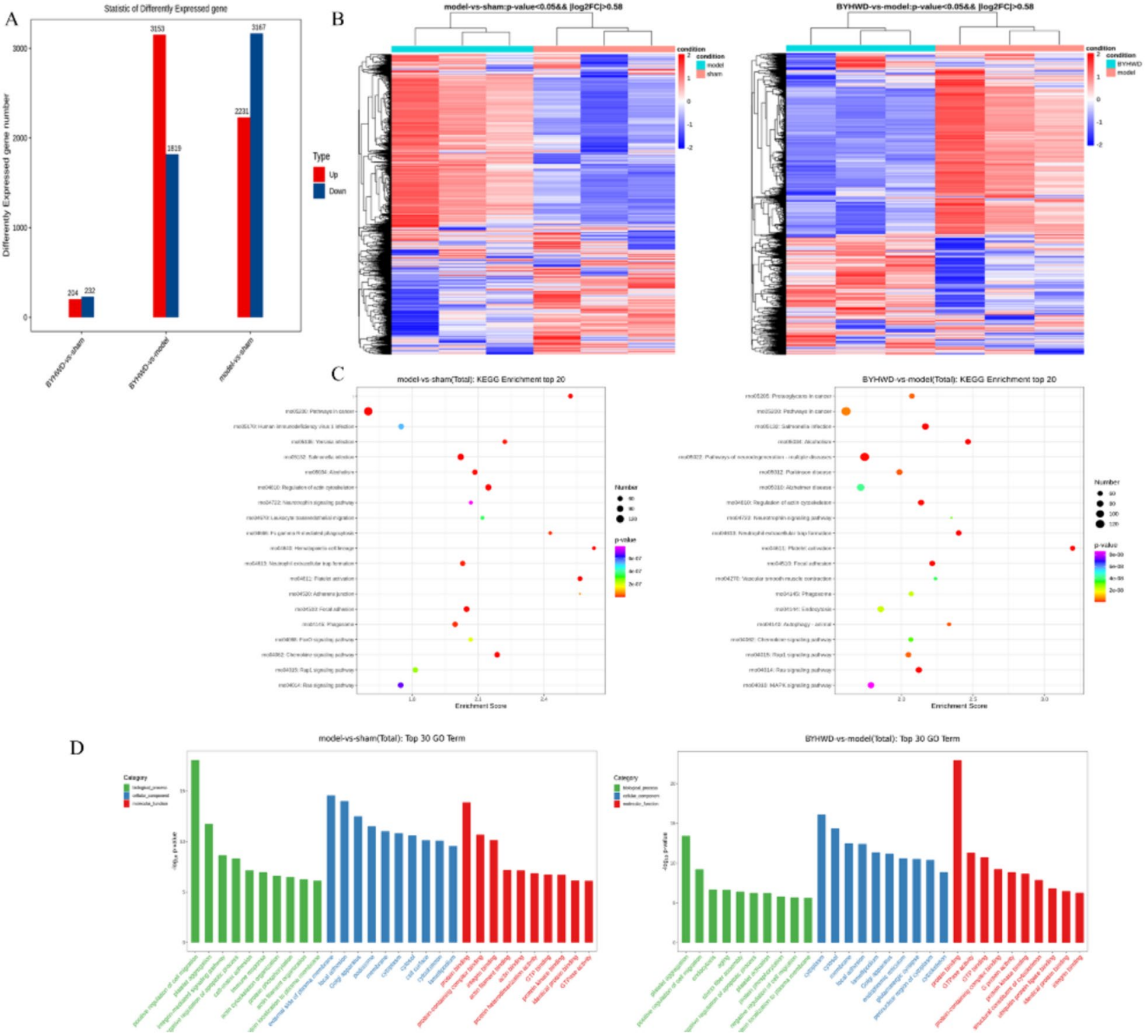
The platelet transcriptome reveals the platelet phenotype affected by BYHWD in MI rats. **a** Differential gene number. **b** Hierarchically clustered heatmap of DEGs (false discovery rate [FDR] < 0.01). The rows describe a gradation of gene expression on a blue (low) to red (high) scale. The columns describe the sample. n = 3 in each group. **c** Enriched signaling pathways for DEGs. Each point stands for a pathway; the x-axis presents the enrichment score, showing that the degree of each pathway is over- or underrepresented of the ranked list of DEGs, normalized to account for differences in gene set size and in correlations between gene sets and the expression dataset. Colors represent the *P* value, and sizes represent the gene number. **d** Gene ontology analysis of cellular components, molecular functions, and biological processes of DEGs

**Fig. 8:**
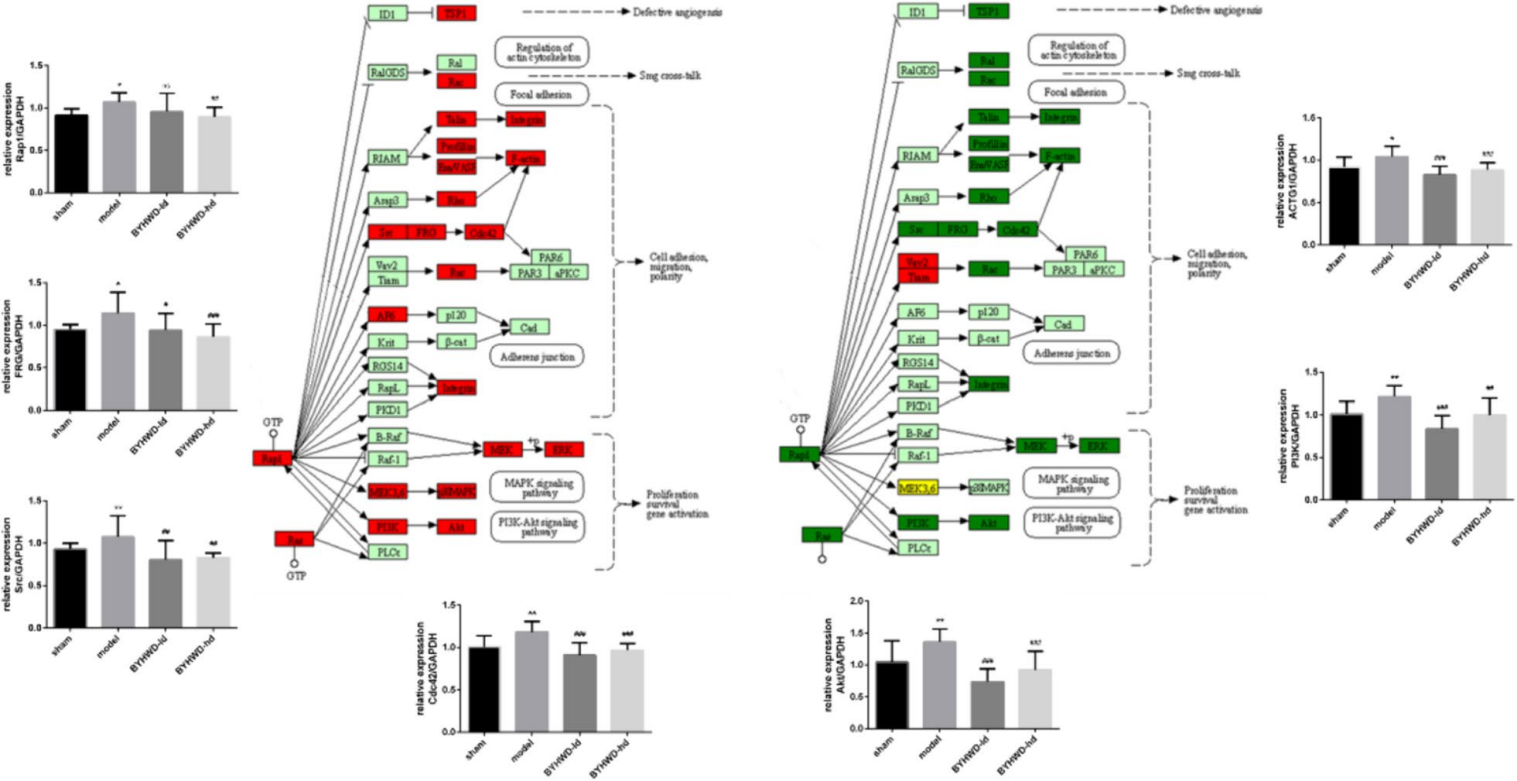
BYHWD alleviated MI-induced platelet activation by downregulating Rap1 signaling. Quantitative real-time PCR analysis of Rap1, FRG, Src, CDC42, Akt, PI3K, and ACTG1. ^*^P < 0.05 and ^**^P < 0.01 when compared to the sham group; ^#^*P* < 0.05 and ^##^*P* < 0.01 when compared to the model group; Data are presented with mean ± SD; n = 6 in each group. Rap1: Ras-related protein Rap-1; FRG: ARH/RhoGEF and pleckstrin domain protein 2; Src: nonreceptor tyrosine kinase; CDC42: cell division cycle 42; Akt: AKT serine/threonine kinase; PI3K: phosphoinositide-3-kinase; ACTG1: actin gamma 1

**Fig. 9:**
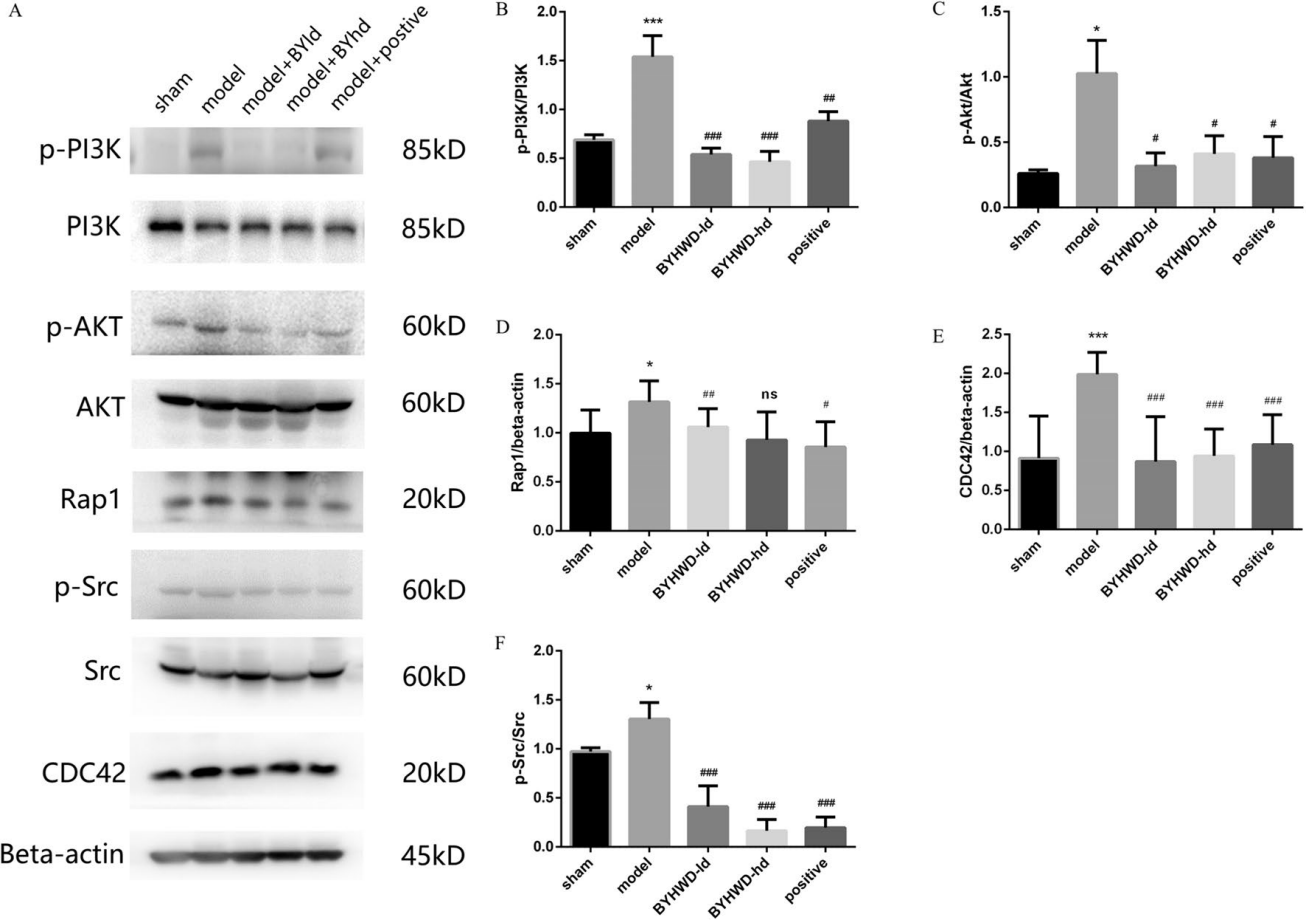
Effect of BYHWD on the expression of proteins Rap1, CDC42, PI3K, Akt, and Src in platelets activated by MI. **a** Representative blots. **b** Quantification of PI3K protein phosphorylation . **c** Quantification of Akt protein phosphorylation. **d** Quantification of Rap1 protein. **e** Quantification of CDC42 protein. **f** Quantification of Src protein phosphorylation. All data were described with mean ± SD (n = 3). ^*^P < 0.05, ^**^P < 0.01 and ^***^P < 0.001 in contrast to the sham group. ^#^*P* < 0.05, ^##^*P* < 0.01, and ^###^*P* < 0.001 in contrast to the model group

Through a transcriptomic approach, the objective was to screen the key pathways by which BYHWD regulates platelets. Platelet genes of the sham, model, and BYHWD-hd group were compared, and 106 differentially expressed genes (DEGs) were screened out (Fig. 7A, B). By summarizing the enrichment of BYHWD, 86 biological processes of Gene Ontology (GO) together with 319 Kyoto Encyclopedia of Genes and Genomes (KEGG) pathways were acquired. The top 20 pathways in three projects were selected based on q-value (Fig. 7). The results of KEGG pathway enrichment suggested that the mechanism of BYHWD in affecting platelet activation after MI was significantly related to the Rap1 signaling pathway (Fig. 7C). We extracted platelet mRNA from the three groups of samples and obtained DNA by reverse transcription to detect gene expression. The results are shown in the pathway diagram (Fig. 8). Green represents gene downregulation and red represents gene upregulation. Compared with levels in the sham group, the expression of Rap1, Src, ARH/RhoGEF and pleckstrin domain protein 2 (FRG), CDC42, PI3K, Akt proteins were upregulated in the model group but downregulated in the BYHWD group with statistical significance. As shown in Fig. 9, ligation contributed to an increased expression of Rap1, CDC42, p-AKT, p-PI3k, p-Src proteins, which are known to participate in the regulation of mitochondrial adhesion and proliferation. Notably, BYHWD significantly restored all the model-induced alterations.

## Discussion

In clinical practice, echocardiography has been used for the assessment of cardiac function and structure. Furthermore, EF as well as FS are key markers of contractility in basic and clinical studies [20]. The heart acts as a pump in the circulatory system. Therefore, SV also plays a basic role in evaluating cardiac function [21]. CM aging has been a pivotal risk factor for fibrosis. Telomere shortening, oxidative stress (OS), along with mitogenic signals are critical in aging [22]. Cell death could be trigged by critically short telomeres. In ischemic injuries due to MI, the rate of shortening may be accelerated in cells subjected to OS/damage [23]. Telomere targeted gene therapy specifically aimed in the improvement of heart function, decreasing scar formation, attenuated the gene expression related with inflammation or proliferation, increased tissue remodelling and potentially regeneration, leading to increased survival rate by 17% compared to controls [18, 24]. In this study, we verified the impacts of BYHWD in MI rats, and BYHWD preserved the parameters of cardiac function, including EF, FS and SV, and alleviated the damage in cardiac tissues along with fibrosis, concomitant with increased telomere expression.

Pharmacological studies indicated that suppression of platelet function is essential in MI. Heart protection effects were associated with suppression of platelet hyperreactivity. A systematic search identified 1583 articles included 10,323 patients in the meta-analysis. Results showed that mean platelet volume, platelet distribution width, platelet aggregation rate, platelet P selectin, fibrinogen, plasminogen activator inhibitor-1, thromboxane B2, 6-keto-prostaglandin F1α, and TXB2/6-keto-PGF1α were higher in the blood stasis syndrome in coronary heart disease group than in the non-BSS patients with CHD group. Regulating platelet reactivity in CHD with qi deficiency and blood stasis syndrome is an important therapeutic strategy [25]. This study found an interesting phenomenon that BYHWD could regulate the excessive repair of myocardial ischemia caused by MI and could simultaneously affect platelet activity after ischemia and prevent thrombosis after vascular injury. In this process, the hyperresponsiveness, aggregation, and adhesion function of platelets were inhibited, but the mitochondrial respiration ability of platelets could be enhanced by BYHWD. More granule release was observed in the BYHWD group under electron microscopy scanning, indicating that BYHWD could enhance the secretion function of platelets, which released active substances to play a role in enhancing cardiac function.

An acute inflammatory response could be provoked by myocardial injury, with the aim of facilitating the adaption to the stressful condition and the restoration of functions. For unresolved tissue injuries, the parainflammatio006E might be induced by tissue-resident macrophages secreting chemokines to recruit leukocytes as well as plasma proteins to the injury area, resulting in a sustained inflammatory response [26]. In persistent tissue malfunction, parainflammation will deteriorate to chronic inflammation, leading to the damages in collateral tissues. Activation of classical neurohormonal systems or haemodynamic overload may make MI ongoing and contribute to deterioration [27]. In physiological conditions, activation of the coagulation system depending on the inflammation induced by a process termed immunothrombosis usually acts as a part of the host response through an interplay between innate immune cells and platelets, with the aim of preventing the systemic spread of platelets in the bloodstream [28]. As highly specialized cells, platelets ensure hemostasis by constantly patrolling the vasculature. They respond to a small amount of agonists and rapidly transit from the anti-adherent to the adherent state to facilitate thrombosis (integrin inside-out activation) in the injured places [16, 29]. The disturbances of the balance in signaling may lead to unwanted platelet activation (thrombosis)/a higher risk of bleeding.

Platelets have a unique metabolic phenotype, exhibiting a high efficiency coupling to mitochondrial ATP production but a low capacity in respiratory reserve [30]. Platelets are recognized as the most active circulating cells in metabolism [31, 32]. Metabolic flexibility is necessary for the transition from the resting state to the activated platelet. Several studies have shown that mitochondria are also related to lipid metabolism, as well as the control of inflammatory responses in addition to ATP synthesis [33]. Mitochondria could regulate the platelet’s prothrombotic function via ATP production & redox signaling. However, platelet mitochondrial dysfunction (MtD) results in decreased ATP production, the impaired Ca^2+^ buffering, and the formation of mitochondrial permeability transition pore (mPTP), together with the additional production of reactive oxygen species (ROS) [34]. Interestingly, as a key signaling event during cell death, mPTP has played a role in platelet activation [35]. Mitochondrial changes may lead to platelet abnormalities. Several pathways of platelet activation have been found to be associated with MtD. MtD acts as a positive feedback, contributing to the formation of procoagulant platelets [36, 37]. It exerts a key effect on platelet activation, which is not linked with platelet aggregation in the early stages [38, 39]. Furthermore, it is also critical for platelet procoagulant activity, promoting the hemostatic response to vascular injuries, but predisposing vessels to thrombotic complications [40]. Several pathways related to MtD have the potential to regulate platelet procoagulant activity through the externalization of phosphatidylserine, such as calcium-dependent pathways and apoptosis-dependent pathways, et al. [41]. In this study, it was found that BYHWD could improve platelet mitochondrial respiration ability and increase basal respiration and maximum respiration values, indicating that BYHWD could improve MtD, thereby affecting abnormal activation caused by mitochondrial disorder. The PI3K/Akt signaling pathway that we focused on in the genetic test may be the signaling pathway responsible for the damaged mitochondrial clearance process. Specific mitochondrial regulation is the focus of our further research.

To explore the targets of BYHWD that act on platelets, a transcriptomics method was chosen to investigate the genetic changes among MI rats and reveal the potential mechanism of BYHWD. Analysis indicated that BYHWD mainly regulated platelet activation, adherens junctions, and focal adhesion via the forkhead box O signaling pathway, chemokine signaling pathway, the Rap1 signaling pathway and the Ras signaling pathway. Rap1, Src, CDC42, PI3K, and Akt were detected and primarily verified in this study. Studies have shown that RAP guanosine triphosphatase (GTPase) is an abundant signaling molecule in the platelet/megakaryocyte lineage and an important regulator of cell adhesion. RAP GTPases, particularly the Ras-related protein Rap-1A (Rap1a) and the Ras-related protein Rap-1B (Rap1b), are pivotal molecular switches for platelet activation and adhesion in injured places [42]. Rap1 signaling exerts a pivotal effect on platelet integrin activation as well as thrombopoiesis. The loss in Rap1b significantly reduces the secretion of α-granule and the activation of the cytoskeletal regulator Rac family small GTPase 1 [42]. As a major convergence point in platelet signaling, Rap1 could result in talin-1 binding to the cytoplasmic domain of integrin β, the activation of the integrin, platelet aggregation, as well as efficient hemostasis [43]. The small GTPases of the RAP subfamily are highly expressed in platelets and play critical regulatory roles in cell adhesion, cytoskeleton remodeling, as well as mitogen-activated protein kinase signaling. Derived from megakaryocytes in the bone marrow, platelets are a major component circulating in the blood, playing a critical role in thrombosis, hemostasis, inflammation, as well as wound healing [44]. As demonstrated in vitro/vivo, mitochondria could affect the metabolic functions of their own cells together with neighboring cells, attributed to their capacity to translocate from one cell to the other [45, 46]. A study showed how intercellular transfer of mitochondria modified the behavior of recipient cells in wound healing progress, which proved the role of platelet mitochondria in activating the proangiogenic activity of mesenchymal stem cells [47]. As a small GTPase of the Ras superfamily, Rap1a connects extracellular signals with intracellular responses [48–50]. It plays a critical role in mediating the effect of the advanced glycation end products and its receptor signaling on downstream protein expression to exert an influence on cellular events related to myofibroblast transition as well as OS [51].

It should be clear that this study confirmed that BYHWD inhibited platelet coagulation and hemostatic activities and reduced the risk of thrombosis recurrence, but at the same time, platelet mitochondrial function and number increased after administration, suggesting that BYHWD facilitates platelet mitochondria wound-healing capability and the potential mechanisms include mitochondrial transfer and metabolic remodeling. Some monomer components in BYHWD have been reported to regulate platelet function. Paeoniflorin improved the morphology / cell viability of human umbilical vein endothelial cells under glucose fluctuation, decreased the rate of platelet aggregation, CD62p expression, as well as ROS concentration, but increased the level of glutathione peroxidase [52]. In the clot retraction test, the volume of precipitated serum can reflect the ability of platelets to contract. Platelet contractile protein causes platelets to protrude from pseudopodia, and attached to the fibrin bundle to form a clot. In the platelet aggregation model induced by shear stress, the inhibitory effect of paeoniflorin on von Willebrand Factor-platelet glycoprotein Ib interaction was significantly. Paeoniflorin treatment (10 mg/kg) for rats significantly prevented arterial thrombosis in vivo without any prolonging effect on bleeding or clotting time [53]. Moreover, hydroxysafflor yellow A attenuated the decreases in the level of platelet cyclic adenosine monophosphate, the activity of protein kinase A, or the expression of receptor γ that activated by peroxisome proliferator. Besides, it also attenuated platelet count in the plasma along with ADP-mediated platelet aggregation. Additionally, it suppressed the expression levels of adhesion molecules together with proinflammatory cytokines among platelets [54]. This study demonstrated the potential of BYHWD to attenuate MI-induced platelet hyperreactivity, which is associated with platelet aggregation and adhesion, clot retraction, increased expression of p-selectin by regulating Rap1 signaling and reducing levels of inflammatory factors, directly providing references for BYHWD treatment in clinical practice.

## Conclusion

BYHWD reduced inflammation and platelet activation via the PI3 kinase/Rap1/integrin α(IIb)β(3) pathway and improved heart function after MI.

### Supplementary Information

Supplementary Material 1. Graphical abstract.

Supplementary Material 2. Table of primers for RT-PCR.

Supplementary Material 3. Identification of serum components after BYHWD treatment.

Supplementary Material 4. The content of NTpro-BNP in the serum.

## Data Availability

The datasets used or analyzed throughout this study are available from the corresponding author upon reasonable request.

## Abbreviations

ACTG1: Actin gamma 1
ADP: Adenosine diphosphate
Akt: AKT serine/threonine kinase
ATP: Adenosine triphosphate
BSA: Bovine serum albumin
BYHWD: Buyang Huanwu decoction
BYHWD-ld: Low dosage of Buyang Huanwu decoction
BYHWD-hd: High dosage of Buyang Huanwu decoction
CDC42: Cell division cycle 42
CMs: Cardiomyocytes
CVD: Cardiovascular disease
DAPI: 4',6-Diamidino-2-phenylindole
EF: Ejection fraction
FCCP: Carbonyl cyanide *p*-trifluoromethoxyphenylhydrazone
FRG: ARH/RhoGEF and pleckstrin domain protein 2
FS: Fractional shortening
GTPase: Guanosine triphosphatase
H&E: Hematoxylin and eosin staining
HPLC: High performance liquid chromatography
HR: Heart rate
IL-6: Interleukin 6
IL-10: Interleukin 10
IFN-γ: Interferon gamma
KEGG: Kyoto Encyclopedia of Genes and Genomes
LV: Left ventricular
LVAWs: Anterior systolic wall thickness
LVEDd: Left ventricular end‐diastolic wall thickness in diastole
LVIDd: Left ventricular end diastolic dimension
LVIDs: Left ventricular end-systolic dimension
LVPWd: Diastolic anterior wall thickness
MI: Myocardial injury
mPTP: Mitochondrial permeability transition pore
OCR: Oxygen consumption rate
p-Akt: Phospho-akt serine/threonine kinase
PBS: Phosphate buffer saline
PI3K: Phosphoinositide-3-kinase
p-PI3K: Phospho-phosphoinositide-3-kinase
p-Src: Phospho-nonreceptor tyrosine kinase
PNA: Peptide nucleic acids
PRP: Platelet-rich plasma
Q-FISH: Quantitative FISH
Rap1: Ras-related protein Rap-1
Rap1a: Ras-related protein Rap-1A
Rap1b: Ras-related protein Rap-1B
RNA-seq: Transcriptome sequencing
ROS: Reactive oxygen species
RPKM: Reads-per-kilobase-per million mapped
RT-qPCR: Quantitative real-time PCR
SD: Standard deviation
SEM: Standard error for the sample mean
Src: Nonreceptor tyrosine kinase
SV: Left ventricular stroke volume
TFUs: Telomere fluorescence units
TNF-α: Tumor necrosis factor-α
VEGF: Vascular endothelial growth factor
