# Supplementary figures and images for: Buyang Huanwu decoction ameliorates myocardial injury and attenuates platelet activation by regulating the PI3 kinase/Rap1/integrin α(IIb)β(3) pathway
Source: Chin Med. 2024 Aug 19;19:109. doi: 10.1186/s13020-024-00976-0 (PMC11331649; doi:10.1186/s13020-024-00976-0)

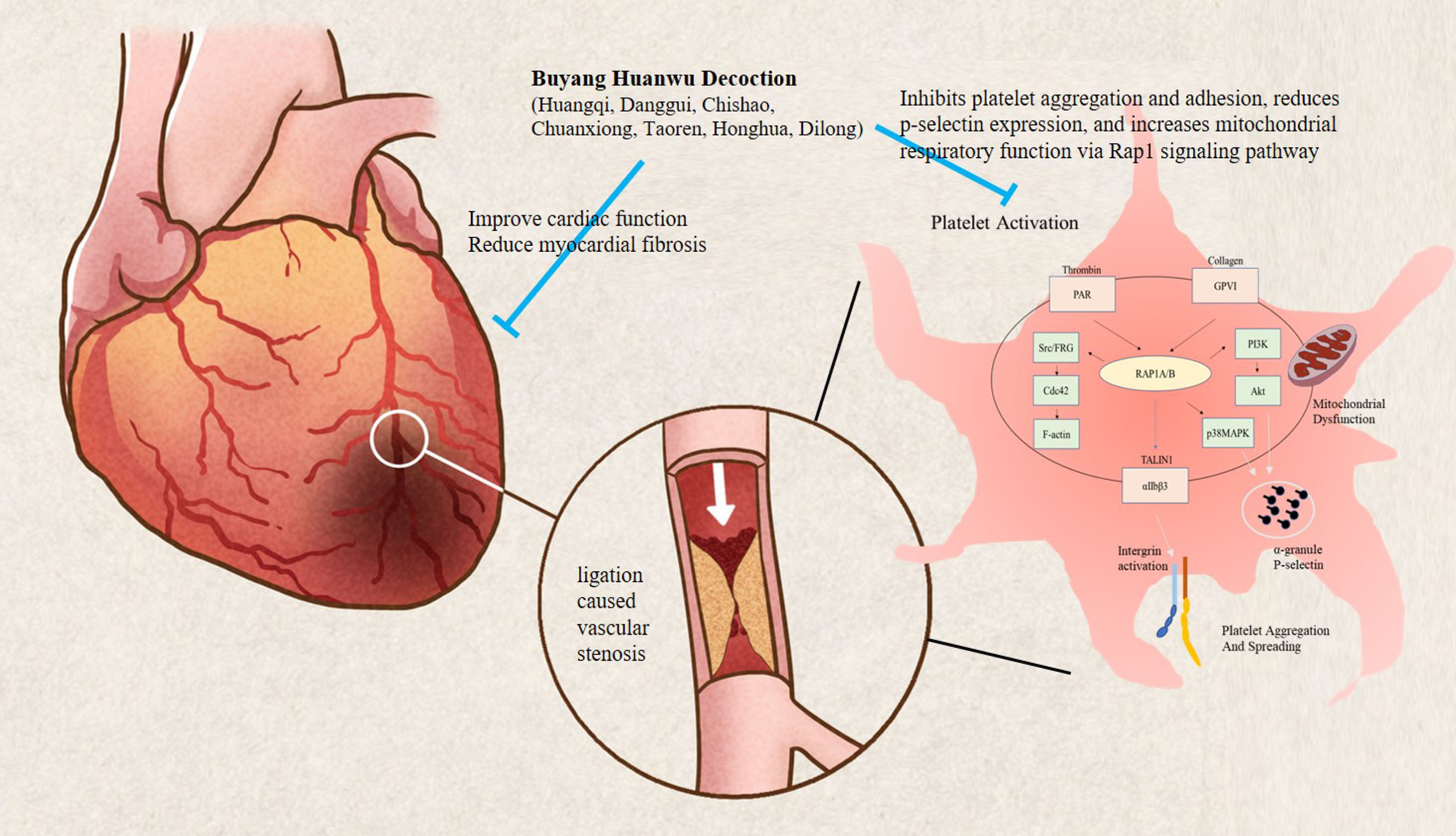

Supplement: Supplementary file 1 — Supplementary Material 1. Graphical abstract. [file 13020_2024_976_MOESM1_ESM.jpg]
