# Supplementary material for: Buyang Huanwu decoction ameliorates myocardial injury and attenuates platelet activation by regulating the PI3 kinase/Rap1/integrin α(IIb)β(3) pathway
Source: Chin Med. 2024 Aug 19;19:109. doi: 10.1186/s13020-024-00976-0 (PMC11331649; doi:10.1186/s13020-024-00976-0)
Supplement: Supplementary file 2 — Supplementary Material 2. Table of primers for RT-PCR. [file 13020_2024_976_MOESM2_ESM.docx]

**Supplemental Material 2**

| Primer |  | Sequence（5’-3’） | Size（bp） |
| --- | --- | --- | --- |
| *Rap1* | F | TATGCTGGAAATCCTGGACACC | 158 |
|  | R | TAACCCGTAAAATCTGTTCTCTCAAG |  |
| *Src* | F | TGTATGCCGTGGTGTCAGAAGAAC | 114 |
|  | R | GCTGGGGTAGCCGCAAGTATTT |  |
| *FRG* | F | AGACGGCACAGTAAGACTCGGAC | 157 |
|  | R | GACCAGGAGAGGACAAAGAAGTAGG |  |
| *Cdc42* | F | AACTCACCACTGTCCAAAGACTCCT | 169 |
|  | R | ACACTCCACATACTTGACAGCCTTC |  |
| *F-actin* | F | AAGAAGAAATCGCCGCCCTCG | 104 |
|  | R | CCCGACGATGGAAGGAAACACG |  |
| *PI3K* | F | CTAAGGAGGAGCACTGTCCGTTG | 92 |
|  | R | GAGATTCAAAGCCATTTTCCCG |  |
| *Akt* | F | ATCGTGTGGCAAGATGTGTATGAG | 197 |
|  | R | GCTGAGTAGGAGAACTGGGGAAA |  |
| *GAPDH* | F | TTCAGCTCTGGGATGACCTT | 129 |
|  | R | TGCCACTCAGAAGACTGTGG |  |

Table. qPCR Primer sequence
