# Supplementary material for: Buyang Huanwu decoction ameliorates myocardial injury and attenuates platelet activation by regulating the PI3 kinase/Rap1/integrin α(IIb)β(3) pathway
Source: Chin Med. 2024 Aug 19;19:109. doi: 10.1186/s13020-024-00976-0 (PMC11331649; doi:10.1186/s13020-024-00976-0)

Supplemental Materials 3. Identification of serum components after BYHWD treatment. A, mix standard, B, blank serum, C, BYHWD serum.


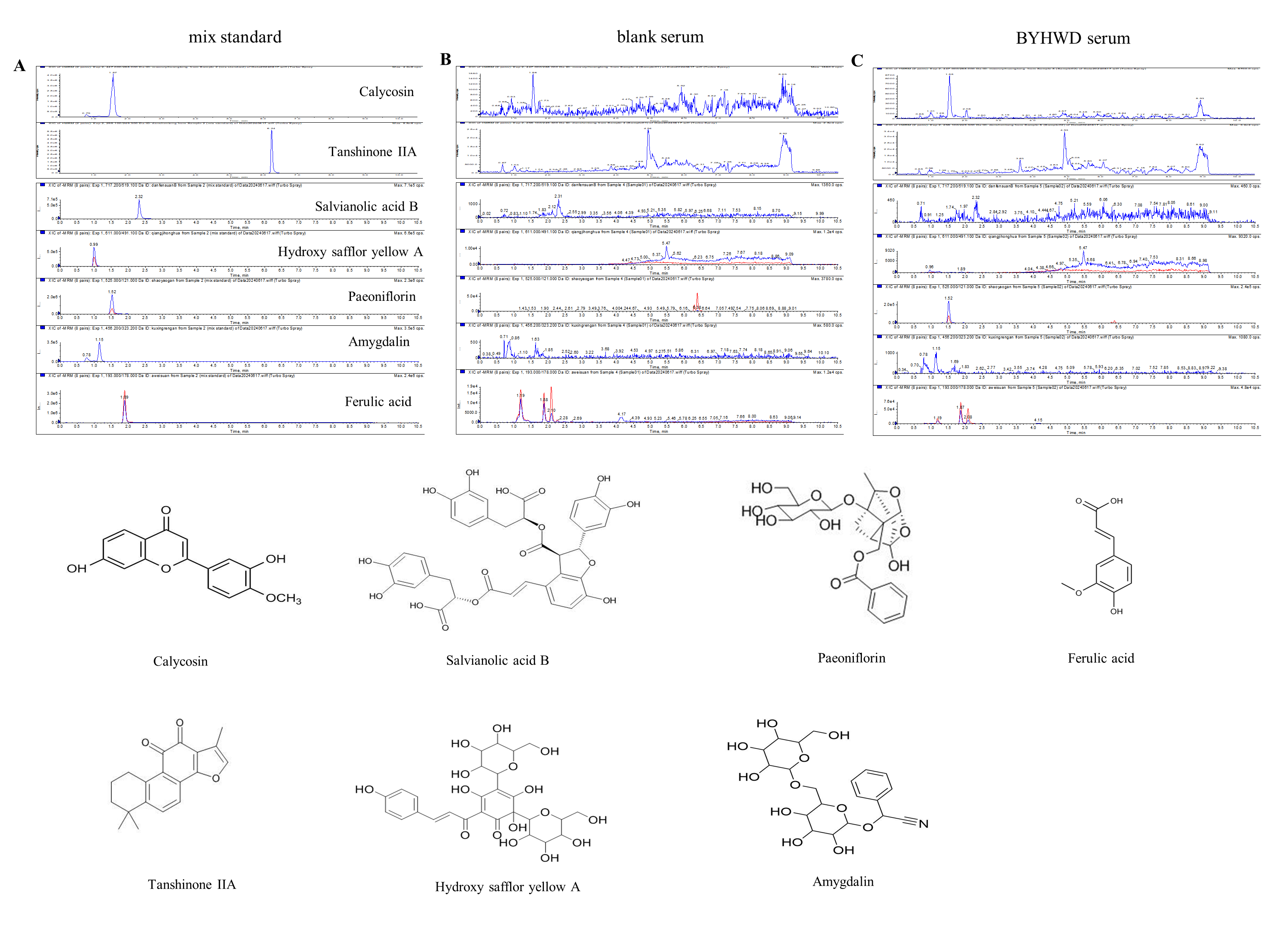

Supplement: Supplementary file 3 — Supplementary Material 3. Identification of serum components after BYHWD treatment. [file 13020_2024_976_MOESM3_ESM.docx]
