# Supplementary material for: Buyang Huanwu decoction ameliorates myocardial injury and attenuates platelet activation by regulating the PI3 kinase/Rap1/integrin α(IIb)β(3) pathway
Source: Chin Med. 2024 Aug 19;19:109. doi: 10.1186/s13020-024-00976-0 (PMC11331649; doi:10.1186/s13020-024-00976-0)

Supplementary Material 4. The content of NTpro-BNP in the serum. All data were described with mean ± SD (n = 6). ^**^*P* < 0.01, in contrast to the sham group. ^#^*P* < 0.05, ^##^*P*< 0.01, in contrast to the model group.


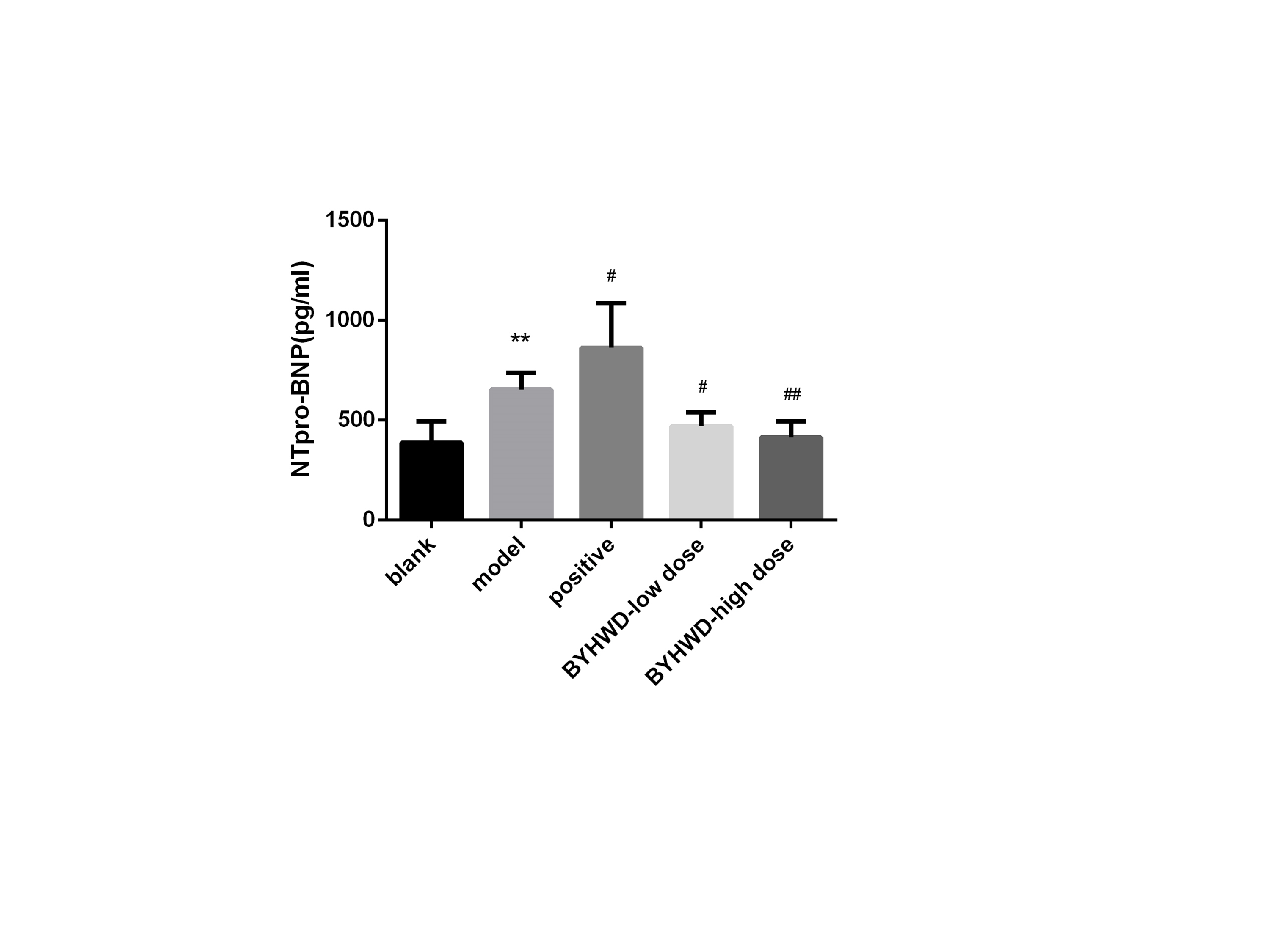

Supplement: Supplementary file 4 — Supplementary Material 4. The content of NTpro-BNP in the serum. [file 13020_2024_976_MOESM4_ESM.docx]
